# Seroprevalence of mycoplasmosis in broiler, layer, and native chickens in Giza, Egypt

**DOI:** 10.1371/journal.pone.0254220

**Published:** 2021-07-12

**Authors:** Saeed El-Ashram, Mahmoud E. Hashad, G. A. Abdel-Alim, Taher Abdelhamid, Heba N. Deif

**Affiliations:** 1 College of Life Science and Engineering, Foshan University, Foshan, Guangdong Province, China; 2 Department of Microbiology, Faculty of Veterinary Medicine, Cairo University, Giza, Egypt; 3 Department of Poultry Diseases, Faculty of Veterinary Medicine, Cairo University, Giza, Egypt; 4 Department of Clinical Medicine, Faculty of Veterinary Medicine, Cairo University, Giza, Egypt; University of Nicolaus Copernicus in Torun, POLAND

## Abstract

We aimed to investigate *Mycoplasma* infections among chicken flocks (Ross, Lohmann and native) in Giza, Egypt, using serological tests, including the slide plate agglutination (SPA) test, hemagglutination inhibition (HI) test, and enzyme-linked immunosorbent assay (ELISA). The slide plate agglutination examination, a serological test, indicated the prevalence of *Mg* and *Ms* infections of 10.9% and 13.2%, respectively. On 91 SPA test positive serum samples for either *Mg* or *Ms*, a passive hemagglutination/hemagglutination inhibition (HI) test was performed. The SPA and HI test findings were found to be comparable. On 90 SPA test positive samples, an ELISA was performed using commercial kits for *Mg* and *Ms* serodiagnosis. According to the ELISA data, only 83.33% and 18.88% of SPA test positive samples were confirmed as positive for *Ms* and *Mg* infections, respectively. The prevalence increased to 84.44% and 77.77%, respectively, when suspected samples were deemed positive.

## Introduction

Avian mycoplasmosis is one of the diseases that have a negative impact on the health and productivity of domestic chickens. *Mycoplasma gallisepticum* (*Mg*) and *Mycoplasma synoviae* (*Ms*) are the major pathogens that cause the disease. *Mycoplasma* infection induces significant economic losses in poultry by reducing; body weight gain, meat quality, feed conversion rate; in broilers, causing a significant decline in egg output in layers, and increasing embryo mortality in breeders [1]. *Mycoplasma* propagation is transmitted both horizontally and vertically in poultry flocks, with the former facilitated by intense industry and stress causes [2]. *Mycoplasma* is a genus of prokaryotes belonging to the Mollicutes family, which includes the smallest recognized self-replicating prokaryotes. *Ms* induces infectious synovitis or mild upper respiratory disease in chickens, while *Mg* triggers chronic respiratory disease in chickens and infectious sinusitis in turkeys. Clinical specimens of *Mycoplasma* species are difficult to cultivate. This is attributable to their meticulous disposition, close links to their hosts, and slow growth on artificial media. Clinical signs of *Mycoplasma* infection can be detected, and *Mycoplasma* can be isolated and identified by culturing on *Mycoplasma* media [3]. Biochemical, serological, or molecular tests are used to identify *Mycoplasma* isolates, as well as serological analysis of host sera using the slide agglutination test (SAT), hemagglutination inhibition (HI) test, or enzyme-linked immunosorbent assay (ELISA) [4]. Nonetheless, since the pathogen has multiple strains, culture-based diagnosis is inadequate for mycoplasmosis [5]. Where concurrent infections are reduced and optimal environmental conditions are provided, controlling the clinical manifestations of *Mycoplasma* infections is easier. Serological examinations are crucial for assessing and tracking the infection status of chicken flocks with *Mycoplasma* [6, 7]. Serodiagnosis is one of the most efficient and accurate approaches for deciding whether or not birds have been subjected to numerous infectious agents. ELISA has been found to have better sensitivity and precision than SAR, although SAR is used as a screening test. Although HI has a high specificity, it is not commercially available and has a low sensitivity. Recently, ELISA with high sensitivity and specificity has become available [8]. The most commonly used tests are slide agglutination, ELISA, and HI, but others have been found, including radioimmunoassay, microimmunofluorescence, and IP assay. Poultry companies that use ELISA technology to screen large quantities of serum samples for virus antibodies can find this assay useful for *Mycoplasma* testing. Serological tests in general can lack accuracy and/or sensitivity; they are better used for flock tracking rather than individual bird research. Diagnosticians who choose to use those instruments should first determine the test’s sensitivity and specificity in their own lab [9]. The aim of the present study was to use the SPA test, HI test, and ELISA to screen broiler, layer, and native chicken flocks of various breeds and ages for serodiagnosis of chicken mycoplasmosis.

## Materials and methods

### Chickens and serum samples

The Department of Microbiology, Faculty of Science, Cairo University approved all animal experiments (CU-04-20), which adopted all national standards for the use of animals in scientific testing, and the basic procedure defined by the OIE. Blood samples from a conscious bird are suitable for diagnostic or testing purposes, according to the AVMA Guidelines for Animal Euthanasia (2020 Edition). In this study, three chicken breeds (Ross (broiler), Lohmann (Layer), and native (baladi)) were investigated. Samples were collected from clinical suspected birds. A total of 1000 blood samples were drawn from the wing veins or during the slaughter of chickens (666 Ross, 95 Lohmann, and 239 native). By centrifugation at 900 x g, serum was isolated from blood, aliquoted into clean tubes, and frozen at -20 ⁰C until required.

### *Mycoplasma* antigens and antisera

The stained *Mg* and *Ms* antigens supplied by Intervet International B.V. (Netherlands) were used in the SPA examination. Antisera against *Ms* and *Mg* were purchased from Animal Health Service Ltd. (Netherlands). Antigens for *Mycoplasma* HI were prepared from *Mg* and *Ms* using the protocols suggested by Cho et al. 1976 [10]. After washing the bacterial harvest, the pellet was resuspended in the glycerine buffer (50.0% Cho buffer and 50.0% glycerine) to achieve a hemagglutination titer of 1:32 or 1:64. When the pellet was mixed with the glycerol buffer at a ratio of 2.0% of the total culture volume of *Mg* and 0.5–1.0% of the total culture volume of *Ms*, this titer was normally produced. The antigen was divided into 2.0 mL aliquots and kept at -70°C.

### Slide plate agglutination (SPA)

Before use, antigens and sera were warmed to room temperature. Thirty microliters of each serum sample was combined in a circular pattern with an equivalent amount of mycoplasma antigen inside 4 cm^2^ squares on a ruled glass plate. The plate was shaken for 5 s. The plate was shaken again for 5 s at the end of the first minute, then read 55 s later. A positive reaction was suggested by the forming of distinct clumps, which normally began at the mixture’s periphery. Positive and negative control sera were analyzed concurrently with the testing of tested samples [5].

### Hemagglutination inhibition (HI)

The HI titers were calculated as the reciprocals of the highest dilutions which resulted in a hemagglutination sheath on the bottom wall of the well. The HI titer of each sample was the highest serum dilution exhibiting complete inhibition of hemagglutination as indicated by formation of a loose button and flowing of cells when the plate was tilted. Antigen titration and HI were carried out following the protocols of USDA (1984) and OIE (2018).

### Enzyme linked immunosorbent assay (ELISA)

The presence of distinct antibodies in the sera of tested chickens was used to diagnose *Mg* and *Ms* infection using commercial indirect ELISA kits (Synbiotics Corp., CA, USA). The manufacturer’s guidelines were followed precisely. The plates were read using an ELISA reader at 405 nm. For MG, samples with OD values less than 0.2 (titer = 0) were considered negative and those with OD values greater or equal to 0.6 (titer = 744 or greater) were considered positive while samples with OD values between 0.2 and 0.599 (titer = 149 to 743) were considered probable (suspect). Concerning *Ms*, samples with OD values less than 0.3 (titer = 0) were considered negative and those with OD values greater or equal to 0.6 (titer = 744 or greater) were considered positive while samples with OD values between 0.3 and 0.599 (titer = 270 to743) were considered probable (suspects).

## Results

### Prevalence of *Mg* and *Ms* by serum slide plate agglutination

Out of 1000 tests, 109 were positive for *Mg* infection (10.9%), and 132 were positive for *Ms* infection (13.2%), as seen in Table 1. Native chickens had a higher frequency of *Mg* and *Ms* illnesses, with rates of 34.72% and 28.03%, respectively. Both *Mg* and *Ms* infections were observed in 2.25% and 5.40% of Ross chickens, respectively, and 11.57% and 27.36% of Lohmann chickens.

**Table 1:** Prevalence of *Mg* and *Ms* infection in tested chickens as indicated by serum slide plate agglutination.

Breed	No. of samples*	*Mg* positive samples		*Ms* positive samples	
		No.	%	No.	%
**Ross**	666	15	2.25	36	5.40
**Lohmann**	95	11	11.57	29	27.36
**Native**	239	83	34.72	67	28.03
**Total**	1000	109	10.9	132	13.20

* The number of samples from each breed was chosen depending on the population in the test field.

### Age-related prevalence of chicken mycoplasmosis by slide plate agglutination test

**Fig 1:**
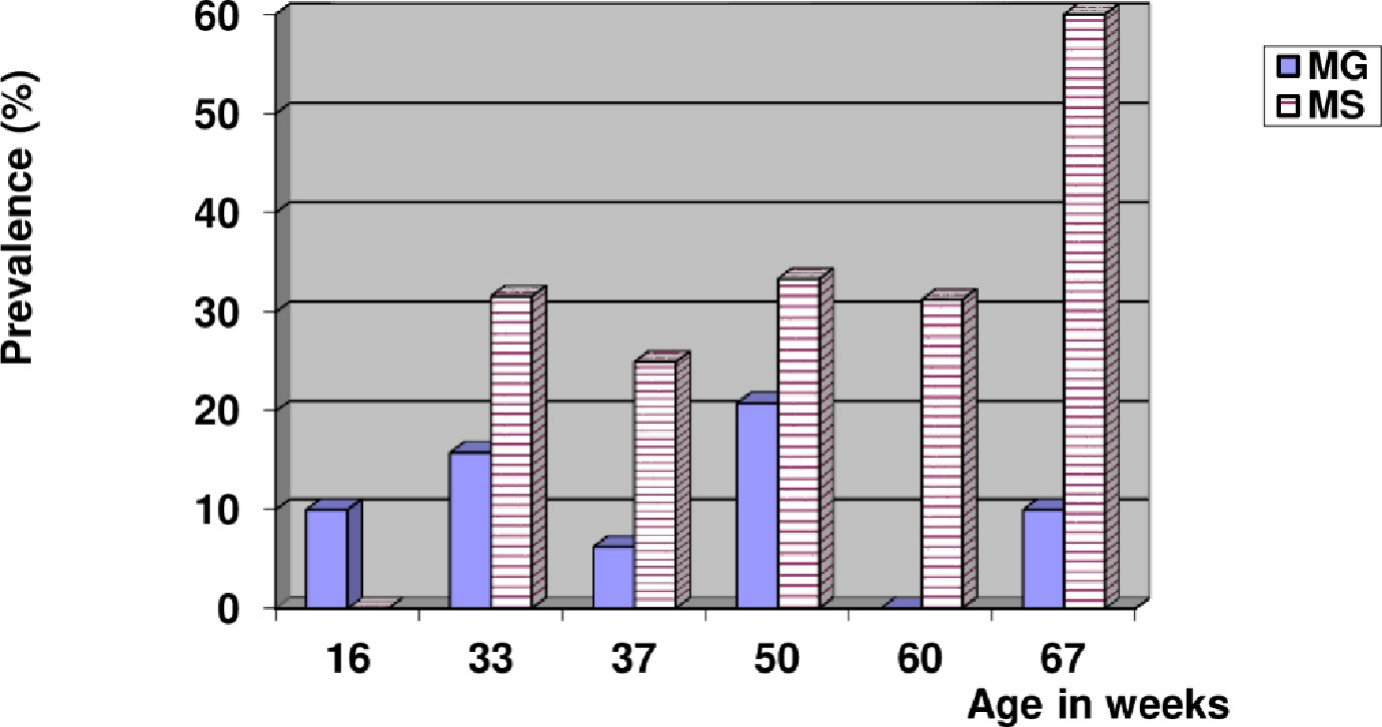
Age-related prevalence of mycoplasmosis in Lohmann layers by slide plate agglutination test.

**Fig 2:**
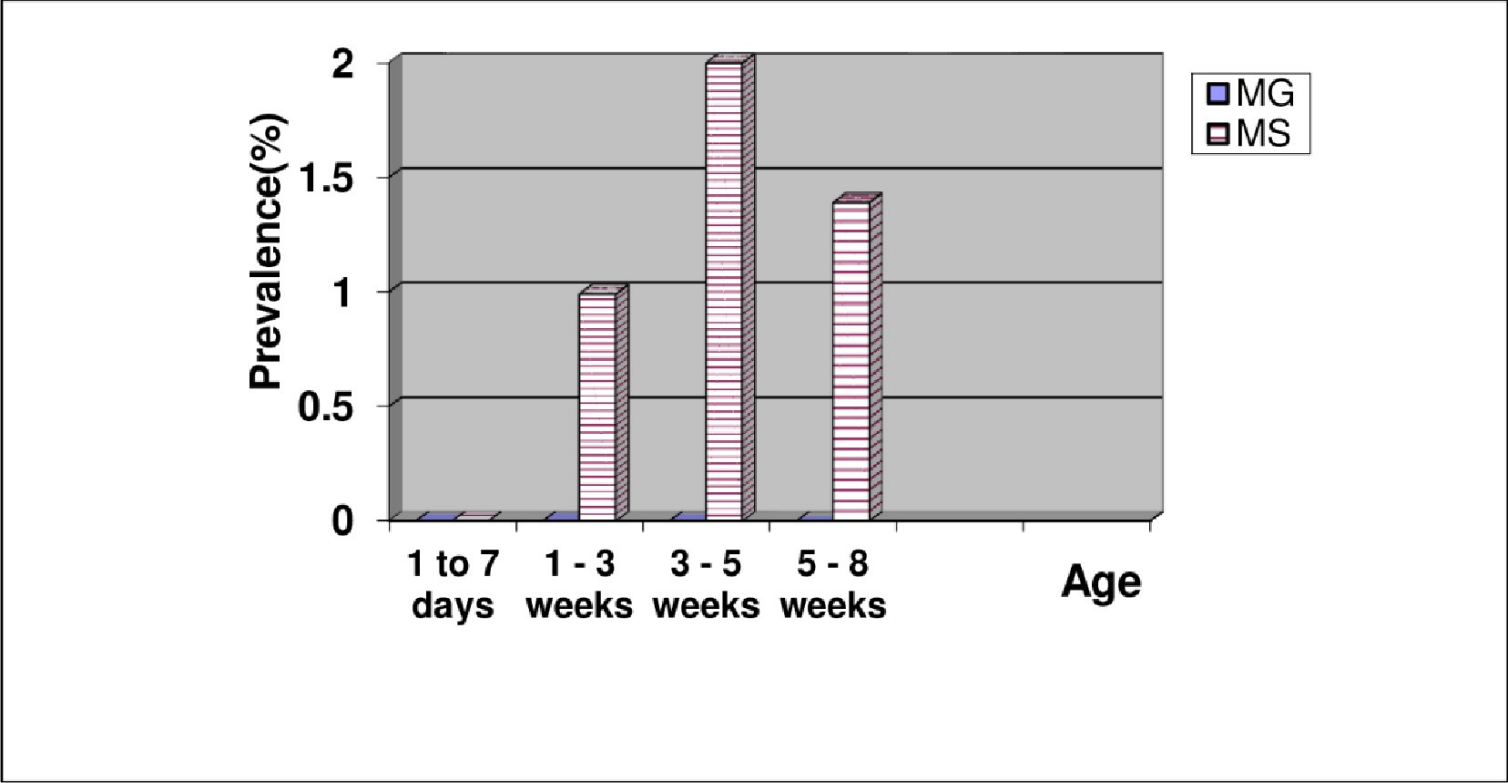
Age-related prevalence of mycoplasmosis in Ross broiler chickens by slide plate agglutination test.

**Fig 3:**
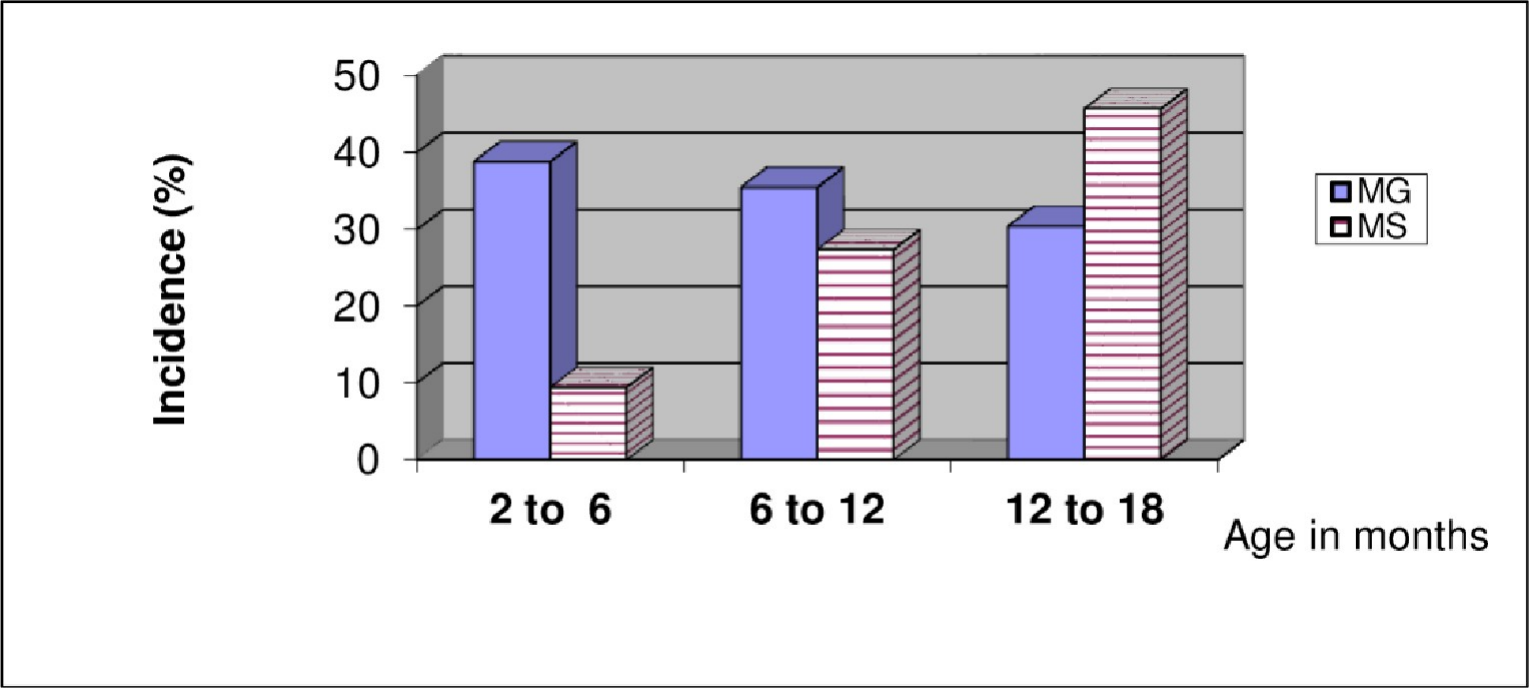
Age-related incidence of mycoplasmosis in native chickens by slide plate agglutination test.

Figs 1–3 illustrate the prevalence of mycoplasmosis, as indicated by the results of the SPA test, among different breeds of chickens investigated in the present study. Different age ranges are represented in the tables in comparison with the frequency of *Mg* and *Ms* infection. Fig 1 shows that the youngest ages yielded the lowest percentage of positivity within Lohmann layers. In the case of *Mg* infection, no consistent relationship between age and infection occurrence was found, and the same was found in the case of mixed infection. *Ms* infection was more prevalent in older birds, with a 60% prevalence rate among 67-week-old birds, and comparable findings were found in Ross and native breeds (Figs 2 and 3). In terms of total prevalence, *Ms* infection was more common in the Lohmann and Ross breeds, whereas *Mg* infection was more common in the native breed.

### Hemagglutination inhibition (antibody titers)

*Mg* and *Ms* plate agglutination positive samples tested positive for HI utilizing the HI-*Mg* and HI-*Ms* antigens. Both antigens could hemagglutinate chicken RBCs in the antigen control wells (without serum). Table 2 shows that no sample had a negative response to the HI test using either the *Mg* or *Ms* antigens. The HI titer; however, differed between tests, varying from 2^3^ to 2^6^ HI units. A clear correlation was observed between the HI titer and the agglutination degree for the same sample as compared with the findings of the SPA examination. It was also discovered that *Ms* antigen produced higher HI titers than *Mg* antigen.

**Table 2:** Hemagglutination inhibition (HI) titers as compared with the slide plate agglutination titers in chicken serum samples against *Mg* and *Ms* antigens.

*Mg* antigens			*Ms* antigens		
No. of serum samples	Slide plate agglutination degree	HI titer	No. of serum samples	Slide plate agglutination degree	HI titer
14	1 to 2	2^3^	13	1 to 2	2^3^
27	2	2^4^	17	2	2^4^
26	2 to 3	2^5^	26	2 to 3	2^5^
24	3 to 4	2^6^	35	3 to 4	2^6^

#### Indirect ELISA

**Fig 4:**
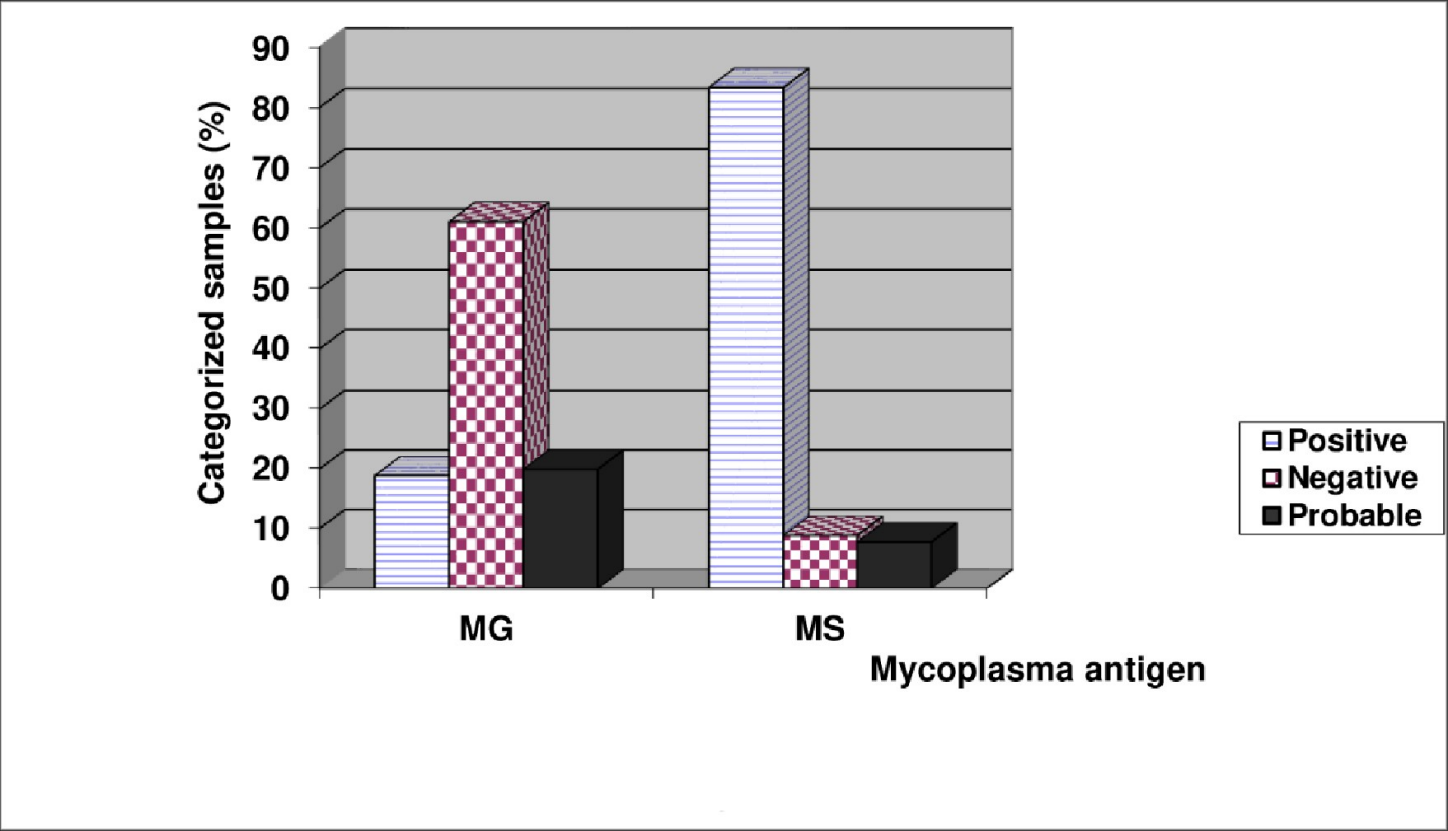
Incidence of *Mg* and *Ms* infection in different chicken breeds tested by ELISA.

Indirect ELISA was used to evaluate 90 serum samples from Ross, Lohmann, and local chicken breeds for the existence of *Mg* and *Ms* antibodies. Table 3 and Fig 4 show the optical density values obtained with various samples. According to the table, the rate of *Ms* infection was higher (83.33%), with 75 of 90 samples having positive O. D. values (0.6). Meanwhile, the percentage of samples with OD values indicating probable (doubtful) results was low in the *Ms* test (7.77%). In contrast, *Mg* infection prevalence (18.88%) was slightly lower than that of *Ms*. Also, the *Mg* test produced further tests with possible findings (20%).

**Table 3:** Results of indirect ELISA for diagnosis of *Mg* and *Ms* infection in different chicken breeds.

O. D. values	*Mg* antigen-coated plate			O. D. values	*Ms* antigen-coated plate		
	No. of samples	Percentage	Interpretation		No. of samples	Percentage	Interpretation
< 0.2	55	61.11%	negative	< 0.3	8	8.88%	negative
0.2–0.6	18	20.00%	Probable	0.3–0.6	7	7.77%	Probable
≥ 0.6	17	18.88%	Positive	≥ 0.6	75	83.33%	Positive
Total	90			Total	90		

Of 90 chicken serum samples, examined for *Mg* and *Ms* infection by ELISA and SPA tests, similar results were obtained in 71.11% and 70% of the samples with *Mg* and *Ms*, respectively. When the probable results were considered positive, the respective percentages increased to 84.44% and 77.77%. When the prevalence of infection was considered with both tests, ELISA prevalence rates were 90% and 38.88% while SPA prevalence rates were 74.44% and 43.33% for *Ms* and *Mg* infections, respectively. As a result, the ELISA findings may be more dependent on than the results of the other two serological studies included in the study. There is no doubt; however, that serum plate agglutination is a fast, low-cost, and reliable test that can be used to initiate a mycoplasmosis serosurvey.

## Discussion

The overall prevalence of *Mg* infection according to the results of the SPA test was recorded to be 10.9% while it was 13.2% for *Ms* infection. Concerning *Mg* and *Ms* infection prevalence in the tested breeds, native chickens showed the highest rates (34.72% and 28.03% respectively). The Lohmann breed showed prevalence rates of 11.57% and 27.36%, and the Ross breed showed the least prevalence rates of 2.25% and 5.4% for *Mg* and *Ms* infection, respectively. The high rates of Mg and Ms infection in native chickens could be attributed to the owners’ rearing and management practices, which include fewer hygienic precautions and therapeutic approaches. Furthermore, the majority of the native flocks used to gather the samples were held in open systems (backyards) with more stressful environmental conditions.

When compared with either native or Lohmann chickens, Ross samples had the lowest rate of infection. However, the majority of the samples came from Ross broilers, where SPA tests are unlikely to reveal positive cases of *Mg* and *Ms*. Serum samples were collected from Lohmann chickens aged 16 weeks and up, which had a higher risk of being positive. Jordan, 1979 [11] stated that the significance of age on tolerance to *Mycoplasma* infection in chickens is unclear when it comes to the age-related occurrence of *Mg* and *Ms* infection. Chickens aged 2 to 20 weeks; however, were found to be vulnerable to experimental infection. The detection of *Ms* infection in Ross chickens aged 5–8 weeks indicates that the antibodies were found in the sera of infected chickens after a period of time (2–3 weeks). Positive responses to both *Mg* and *Ms* antigens were observed in certain Lohmann and native chicken tests, suggesting either mixed infection or cross reactivity. It was reported that serum from *Mg*-infected birds did not react with *Ms* antigen. In contrast, sera from *Ms*-infected birds reacted with the *Mg* antigen. Cross reactions; however, did not pose a concern in other experiments, such as the HI test [12, 13]. Positive SPA test reactions for both antigens may also be called *Ms* infection while mixed infection is unlikely. Other tests, such as HI, can confirm this. In the HI test, only 12 of 18 samples reacted positively with Ms antigens, while 6 reacted positively with both antigens. This supports the finding that cross reactions in the serum SPA test do not represent a problem with the HI test [12, 13]. The *Ms* detection HI test tends to be reasonably specific, and it can be used in combination with the *Mg* detection HI test to differentiate between *Ms* and *Mg* infection. For detecting antibodies against *Mg*, the HI test was suggested as an official test and as the most reliable serological test [14]. The positive response of a sample to a serum plate test when the same sample was negative to an HI test utilizing the same antigen was traditionally due to the bird’s treatment of immunosuppressive drugs including chlorotetracycline in different doses. Early antibodies (IgM agglutinins) may be inhibited to a lesser degree by certain medications than HI late IgG antibodies [15]. The diagnosis of avian mycoplasmosis was achieved using ELISA. The assay was described as a successful test for that purpose [16, 17]. In the present study, concordance rates between ELISA and serum SPA were 71.11% and 70% with *Mg* and *Ms* infection, respectively. When questionable ELISA results are considered positive, the rates rise to 84.44% and 77.77%, respectively. Apart from concordance, *Mg* and *Ms* infection rates with ELISA were 38.88% and 90%, respectively, and with the serum SPA test were 43.33% and 74.44%. This confirms once more the possible cross reaction of serum samples from *Ms*-infected birds with *Mg* antigen in the serum SPA test [12, 13].

As a more precise measure, ELISA may differentiate between false and true positive reactions. ELISA was identified as an easy, effective, and specific assay in related studies, and it was used to track the appearance of specific anti-*Mg* IgG in chicken sera at different intervals after the onset of mycoplasma-induced respiratory disease [18]. Antibodies to MS were also detected using ELISA in the egg yolks of commercial layers as proof of MS infection. The assay showed a prevalence of egg antibody to MS of 78.6%, and it was identified as a sensitive and specific test [19]. When compared to other serologic studies, ELISA was found to be more accurate than HI in confirming Mg infection in fair and exhibition birds. In birds experimentally infected with *Mg* by Ewing et al. [20], there were no significant differences between HI and ELISA.

The *Mg* serum SPA assay cross-reacted with 15% of 195 sera obtained from flocks that were reported positive for *Ms* by culture. Neither the *Mg* ELISA nor the HI revealed any cross-reactions. In populations with a low prevalence of *Mg* infection, the findings revealed no differences between ELISA and HI as confirmatory tests. In a population with moderate levels of *Mg* infection; however, ELISA was superior to HI [20]. Feberwee et al. (2005) [21] reported that ELISA and the rapid SPA test using undiluted serum showed a relatively high number of false-positive results for mycoplasmosis serosurvey. Every serological test, according to the findings, should predict a certain level of false-positive results. Because the prevalence of false-positive results varied between serologic studies, relying solely on one test method is not recommended. Serum samples were diluted in ELISA but not in SPA or HI experiments in this analysis. As a result, the findings of the ELISA may be relied on more than the results of the other two serological experiments included in the analysis. There is no doubt; however, that serum SPA is a quick, low-cost, and reliable test that can be used to initiate a mycoplasmosis serosurvey.
